# Nerve Blocks for Hip Fractures in the Emergency Department: An Opportunity for Growth

**DOI:** 10.5811/westjem.43500

**Published:** 2025-09-25

**Authors:** Robert Allen, Dainis Berzins, Lydia Koroshetz, Chun Nok Lam, Melissa Wilson, Mayra Cruz, Jennifer Huang, Dana Sajed, Thomas Mailhot

**Affiliations:** * Los Angeles General Medical Center, Department of Emergency Medicine, Los Angeles, California; † Cedars Sinai Medical Center, Department of Emergency Medicine, Los Angeles, California; ‡ University of Southern California, Keck School of Medicine, Department of Emergency Medicine, Los Angeles, California; § University of Southern California, Keck School of Medicine, Department of Population and Public Health Sciences, Los Angeles, California

## Abstract

**Introduction:**

Hip fractures are a common reason for presentation to the emergency department (ED) and are associated with significant morbidity. Nerve blocks have emerged as a safe and effective tool to treat pain associated with hip fractures. In this study, we aimed to measure the frequency with which nerve blocks were performed for ED patients with hip fractures. Our secondary aims were to study the demographic and clinical characteristics of patients who received and did not receive a nerve block.

**Methods:**

*:* We performed a retrospective study at a single-center, urban, academic, Level I trauma center. We measured the frequency with which patients received a nerve block. We measured other demographics (age, ethnicity, insurance) and clinical data (comorbidities, Emergency Severity Index, National Emergency Department Overcrowding Scale, and hip fracture type). Lastly, we measured the types of nerve block performed, who performed the nerve block, and any associated complications.

**Results:**

*:* Overall, 17% (36/209) of the studied patients and 14% (36/257) of all patients with an acute hip fracture received a nerve block. Patients who were cared for by ultrasound (US) fellowship-trained physicians were more likely to receive a nerve block compared to patients cared for by non-US fellowship-trained physicians (20/35 vs 16/174; *P*-value < .001).

**Conclusions:**

*:* Nerve blocks were performed for a minority of patients presenting with an acute hip fracture. Patients who are cared for by ultrasound fellowship-trained physicians may be more likely to receive a nerve block than patients cared for by non-ultrasound fellowship-trained physicians in the emergency department.

## INTRODUCTION

Hip fractures are commonly diagnosed in the emergency department (ED). Such fractures are extremely painful and are associated with significant morbidity.^1^,^2^ Emergency physicians caring for such patients are primarily tasked with providing appropriate analgesia, which is associated with improved outcomes postoperatively.^3^ However, traditional analgesic management, often involving intravenous opioids, is associated with oligoanalgesia, delirium, and hypoxia.^4^,^5^ The peripheral nerve block is a novel approach that, compared to standard care, has been found to reduce pain, reduce opioid use, encourage earlier mobilization, and decrease the incidence of pneumonia.^6^ Femoral nerve blocks and fascia iliaca compartment blocks in the ED are both feasible and associated with improved outcomes.^7^–^9^ More recently, the pericapsular nerve group (PENG) block has shown promise as an alternative nerve block to treat hip fractures.^10^–^13^ Professional organizations within the specialties of emergency medicine, orthopedics, trauma surgery, and anesthesia now endorse the use of peripheral nerve blocks.^14^–^17^ Despite this endorsement, the frequency with which they are performed in the ED is unknown. Additionally, it is currently poorly understood what factors influence whether a patient receives a nerve block in the ED.

In this study we aimed to assess 1) the frequency with which nerve blocks are performed for patients with hip fractures in the ED and 2) the demographic and clinical characteristics of patients who received and did not receive nerve blocks. Lastly, we describe the characteristics of the nerve blocks performed.

## METHODS

We conducted a retrospective, cohort study to describe the characteristics and experience of patients who presented to the ED at Los Angeles General Medical Center and were diagnosed with hip fractures. Los Angeles General Medical Center is an urban, academic, tertiary-care center serving the County of Los Angeles, with an annual census of over 150,000 ED visits per year. The primary outcome was to measure the proportion of patients who received a nerve block for treatment of hip fracture. The secondary outcomes were to report the demographic and clinical characteristics of patients with hip fractures who received and did not receive a nerve block. Institutional review board approval was granted for the study with a waiver of patient consent.

Inclusion criteria included adult patients (≥ 18 years of age) with an acute hip fracture diagnosed in the ED. Exclusion criteria included patients who were pregnant, incarcerated, had contraindications to receiving a nerve block (intubated patients, patients with an altered mental status, or presence of severe polytrauma), and patients transferred from another ED. (Appendix A)

We followed best practices for chart review as described by Worster et al.^18^ Blinding of abstractors (DB, LK) was not possible as the two abstractors were study investigators. A list of potentially eligible patients was generated from the *International Classification of Diseases, Rev 10*, (Appendix B) by an investigator (CL). From this list, the date of ED visit, Emergency Severity Index (ESI) score, National Emergency Department Overcrowding Scale (NEDOCS) score, and insurance were generated using hospital administrative data. Manual data abstraction was performed by two investigators (DB, LK). A coding guide was created prior to data abstraction (Appendix A). Abstractors reviewed notes from emergency physicians, orthopedic consult notes, and admission and discharge notes.

We developed a data abstraction form using REDCap (Research Electronic Data Capture tools hosted at University of Southern California)^19^ (Appendix C). The data abstraction form was pilot-tested before use. Discussions were held throughout data abstraction to address questions. Both reviewers received the same random set of 10% study cases for initial training. We calculated inter-rater reliability (Cohen kappa) upon chart review of the first 5% of cases, and the lead author met with both reviewers to discuss data field abstraction methods to synchronize the approach. Upon completing the second 5%, we achieved a kappa value of greater than 0.8. The reviewers then received an evenly distributed set of remaining cases to review independently. For records that were found to be in disagreement from the first 10%, the lead author conducted chart review separately to serve as the tiebreaker.

**Population Health Research Capsule**

What do we already know about this issue?

*Nerve blocks are a safe and effective tool to treat pain associated with hip fractures in the emergency department.*

What was the research question?

*We measured the frequency with which nerve blocks were performed for ED patients with a hip fracture.*

What was the major finding of the study?

*Overall, 17% (36/209) of the studied patients and 14% (36/257) of all patients with an acute hip fracture received a nerve block.*

How does this improve population health?

*The nerve block is an opioid-sparing method to treat pain. Future studies are needed to help improve the rate of nerve blocks performed in the ED.*

Patients were first screened for inclusion and exclusion criteria. We excluded patients if the hip fracture was not acute (based on either radiology interpretation or emergency physician documentation.) Included patients had the following data abstracted: age; sex; ethnicity/race; comorbidities; hip fracture type; arrival method; name of emergency clinician; whether or not a nerve block was performed; who performed the nerve block; type of nerve block performed; and nerve block complication(s). The ESI and NEDOCS scores and insurance information were obtained from hospital administrative data by a third investigator (CL). The NEDOCS score is a validated measurement, collected every two hours at our hospital to monitor ED crowding.^20^,^21^ We used this as a surrogate measure to correlate with the subjective sense of how busy the ED may have felt to the emergency clinician at the time. Nerve block complications were determined by reviewing the ED and admission notes (Appendix A.)

We determined who performed the nerve block by reviewing physician notes, procedure notes, and nursing documentation. A block was considered to be performed by a resident if both a resident and an attending were present for the procedure. This was done to separate blocks that were done independently by an attending. At our institution, most patients are treated primarily by residents, with attendings acting in a supervisory role. Attendings are required to be present for all ultrasound (US)-guided nerve blocks. We defined US-fellowship trained physicians to include fellows currently in an US fellowship and attending physicians who had completed an US fellowship or received Advanced EM Ultrasonography certification. Our center does not have an established protocol for performing nerve blocks in the ED for hip fracture patients. and the decision to perform a block is made by the treating emergency physician. In our experience, all nerve blocks in our department are performed with US guidance, typically using bupivacaine without epinephrine. Our institution does not have a formal nerve block-training program; however, residents and faculty are exposed to periodic didactic and hands-on training as part of the residency educational curriculum.

A priori, we anticipated, based on prior history, that approximately 480 patients would be available for analysis (20 patients/month over 24 months), with 10–20% estimated to receive blocks. Thus, assuming the block prevalence to be 20%, we calculated the precision with which we could estimate the prevalence of blocks within hip fractures, with 95% confidence to be between 16–24%. Demographics and clinical characteristics are reported as the median or count with frequency. We reported characteristics for the total population of patients with hip fractures, patients who received nerve blocks, and patients who did not receive nerve blocks. We used the Wilcoxon rank-sum test to test numeric variables and chi-square and Fisher exact tests to compare demographic and clinical factors by nerve block performed. All analyses were performed in Stata 15 (StataCorp, LLC, College Station, TX) with set to 0.05.

## RESULTS

A total of 347 patients were reviewed for eligibility from August 1, 2021–July 31, 2023 (Figure). There was excellent agreement among reviewers from the first 10% of the sample (K = 0.93). We excluded 138 patients: 90 did not have an acute fracture; 18 were transferred from another facility; and 36 had a contraindication to receiving a nerve block. (Some patients had more than one exclusion criteria.) A total of 36 patients received a nerve block, representing 17% (36/209) of the studied patients and 14% (36/257) of all patients with an acute hip fracture.

Demographics were similar for those patients who received a nerve block and those who did not (Table 1). We found no correlation between the likelihood of receiving a block and patient’s age, sex, ethnicity, language, or insurance.^22^ The median age of all patients in the study was 77.1 years old. Approximately two-thirds of the patients were female. Over 50% of patients in our study were Hispanic/Latino and did not speak English, reflecting the overall demographic of our medical center. Medicare and Medi-Cal were the most common insurances of patients, representing 87.1% of all included patients.

Patients who were cared for by US fellowship-trained physicians were more likely to receive a nerve block compared to patients cared for by non-US fellowship-trained physicians (*P*-value < .001) (Table 2). Ultrasound fellowship-trained physicians performed 55.6% of total nerve blocks. Likewise, patients who did not receive a block were more likely to be cared for by non-US fellowship-trained physicians (91.3%) than by US fellowship-trained physicians (8.7%). A total of 20 blocks were performed by two full-time US fellowship-trained physicians, three part-time US fellowship-trained physicians, and three US fellows. There were 63 non-US fellowship-trained full/part-time physicians involved in the study. Other clinical characteristics were similar between patients who did and did not receive nerve blocks (Table 2). The fascia iliaca block was the most commonly performed nerve block followed by the PENG block (Table 3). Most nerve blocks were performed by emergency medicine residents on clinical shifts, followed by emergency medicine residents on an US rotation, and US fellows.

One potential complication was noted in a patient who received a nerve block and suffered a ventricular fibrillation arrest. However, the arrest occurred hours after the nerve block and was thought by the caring physicians to have been caused by hypoxia after receiving intravenous fentanyl. Although local anesthetic toxicity can cause ventricular arrhythmias, this was thought to be less likely in this case, given the time course and a more likely explanation.

## DISCUSSION

Despite strong evidence supporting peripheral nerve blocks for hip fractures, we found that at our Level I academic trauma center, nerve blocks were performed in a minority of patients. While our findings are limited to our single center, we believe our research should lead to future study to measure the rate of nerve blocks at other centers. We believe this to be an important opportunity for growth within our medical center and the greater emergency medicine community. Our findings are particularly noteworthy given it was conducted at an academic institution where most patients are cared for primarily by residents. We believe residents should be supported to perform nerve blocks in the expectation that they can continue this practice after completing their training.

Patients receiving nerve blocks were more likely to have been treated by US fellowship-trained physicians, despite the fact that US fellowship-trained physicians only represented a minority of the total treating physicians in our study. These results may not be surprising given that nerve blocks are commonly performed using US. A survey conducted in 2022 found that 100% of academic EDs with US fellowships perform US-guided nerve blocks at their institutions.^22^ However, the rate of nerve blocks performed at centers without an US fellowship is still uncertain. Future initiatives to improve the rate of nerve blocks may consider focusing efforts on increasing the rates of nerve blocks performed by non-US fellowship-trained physicians.

There are multiple variables that may serve as potential facilitators and barriers to performing a nerve block. While we did not find any statistical significance other than the presence of an US fellowship-trained physician, it is possible and likely that there were other contributing factors. Prior studies have found that patients at extremes of ages and non-White patients receive different pain treatment modalities.^23^–^25^ Our study was underpowered to explore differences in demographic characteristics. Future studies of larger sample sizes could incorporate this potential factor.

In our study we did not consider the age or experience of the attending physician. Given that US-guided nerve blocks are a relatively newer skill, it may be possible that clinicians who more recently completed residency training may be more familiar with and, therefore, more likely to perform nerve blocks, compared to clinicians who completed residency earlier. This hypothesis could be tested in future studies.

Additionally, based on clinical experience, periods of larger patient volume may influence the likelihood of performing a nerve block. The ED census may be influenced by a variety of factors such as time of day, day of the week, and seasonal variation. Therefore, when an ED is “busier,” clinicians may feel that they have less time to perform a nerve block, which may be considered an optional procedure. While our study was not powered to detect differences in this predictor, it is possible that a larger study could test this hypothesis.

Although the fascia iliaca block was the most commonly performed block during the study time frame, it is possible this may not reflect current or future practice. The PENG is a newer block, first described in the ED literature in 2020.^11^ It is, therefore, possible that as physicians become familiar with this block it may be used more frequently. A 2024 meta-analysis found that the PENG block may have better efficacy than both femoral and fascia iliaca blocks.^10^ Whereas the femoral nerve block was once preferred, none of the patients in our study received this block, perhaps reflecting the practice of our institution. Farrow et al in their review of nerve blocks in a community teaching hospital, similarly found a higher percentage of fascia iliaca blocks compared to PENG blocks, without any femoral nerve blocks performed.^26^ Likewise, Goldsmith et al in their multicenter observational registry, found fascia iliaca/femoral nerve block to be the most commonly performed nerve block.^27^ We would hypothesize that other centers and different time frames of studies may show different preferences for nerve blocks.

## LIMITATIONS

Our study had multiple limitations. First, our study was limited by its retrospective design. It is possible, albeit unlikely, that patients received a nerve block without documentation. It is standard practice that any procedure is documented in the electronic health record. As a safeguard, we reviewed all physician and nursing notes to determine whether a nerve block had been performed. Likewise, it is possible that an US fellowship-trained physician was present for the procedure but not documented. We chose a conservative approach of only stating that an US fellowship-trained physician was present if they were one of the treating physicians or if it was documented that they were present for the block. If US fellowship-trained physicians were present at blocks but not documented, this would further strengthen the statistically significant difference found in our study.

Second, we did not report pain scores of patients who received nerve blocks. This was due to the retrospective nature of our study. Third, the outcomes from our single-center study may likely differ from the outcomes at other institutions. Our medical center is an academic Level I trauma center where most patients are cared for by residents. Centers without residents or US fellowship-trained physicians, such as community hospitals, may perform fewer nerve blocks, especially if physicians are less familiar with the procedure. Future studies could consider studying the rate of nerve blocks for hip fractures, especially across different types of medical centers. Furthermore, the demographics of our patients likely differ from other centers. Future study could explore whether there are differences in the rate of nerve blocks performed in centers with different demographics. Lastly, it is possible that our study was too small to consider possible confounders in the analysis.

## CONCLUSION

Our single-center, academic, retrospective study found that nerve blocks were performed in a minority of patients with hip fractures. Patients who are cared for by ultrasound fellowship-trained physicians may be more likely to receive a nerve block than patients cared for by non-ultrasound fellowship-trained physicians in the emergency department.

## Supplementary Information





## Figures and Tables

**Figure:**
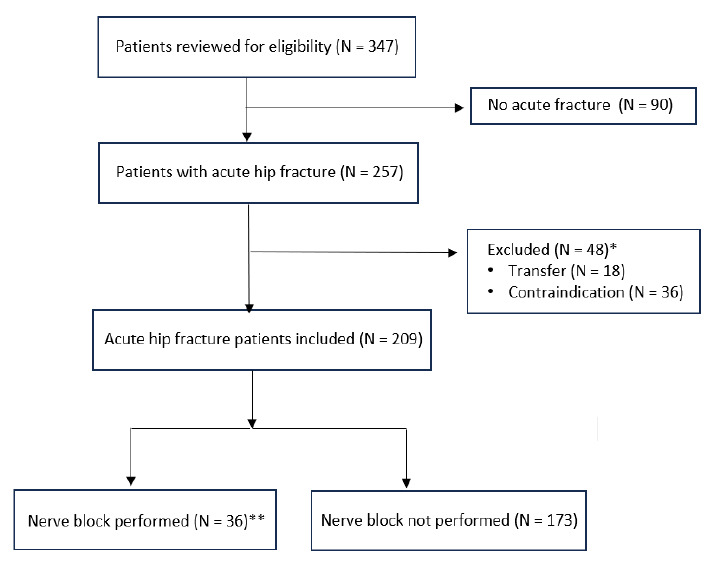
Flow diagram of how patients were chosen to receive nerve blocks for acute hip fractures. *Some patients had multiple exclusion criteria, which is why the value does not total 48. **Represents 17% of patients studied and 14% of total patients with an acute hip fracture.

**Table 1:** Demographics of all patients with a hip fracture, stratified by those with and without a nerve block.

	Total Population (N = 209)	Nerve Block (N = 36)	No Nerve Block (N = 173)	
Variable	N (%) or Median (IQR)	N (%) or Median (IQR)	N (%) or Median (IQR)	P-Value*
Age	77.1 (23.3)	79.7 (23.2)	75.9 (23.1)	.22
Sex				.39
Female	132 (63.2)	25 (69.4)	107 (61.8)	
Male	77 (36.8)	11 (30.6)	66 (38.2)	
Ethnicity				.76
Hispanic/Latino	119 (56.9)	20 (55.6)	99 (57.2)	
Not Hispanic/Latino	87 (41.6)	15 (41.7)	72 (41.6)	
Unknown	3 (1.4)	1 (2.8)	2 (1.2)	
Language				.12
English	92 (44.0)	16 (44.4)	76 (43.9)	
Spanish	86 (41.1)	11 (30.6)	75 (43.4)	
Other	31 (14.8)	9 (25.0)	22 (12.7)	
Insurance				
Medicare	117 (56.0)	22 (66.1)	95 (59.4)	.50
Medi-Cal	65 (31.1)	8 (22.2)	56 (32.4)	.23
Commercial	21 (10.1)	5 (13.9)	16 (9.2)	.40
Self-pay	6 (2.9)	0 (0)	6 (3.5)	.26

* Chi-square, Fisher exact, and Wilcoxon rank-sum tests were performed.

*IQR*, interquartile range.

**Table 2:** Clinical characteristics of all patients with a hip fracture, stratified by those with and without a nerve block.

	Total Population (N = 209)	Nerve Block (N = 36)	No Nerve Block (N = 173)	
Variable	N (%) or median (IQR)	N (%) or median (IQR)	N (%) or median (IQR)	*P*-value*
US Fellowship-trained Physician Present				< .001
Yes	35 (16.7)	20 (55.6)	15 (8.7)	
No	174 (83.3)	16 (44.4)	158 (91.3)	
Hip Fracture type				
Intertrochanteric	117 (56)	20 (55.6)	97 (56.1)	.96
Femoral Neck	67 (32.1)	9 (25)	58 (33.5)	.32
Femoral head	11 (5.3)	4 (11.1)	7 (4.1)	.10
Greater trochanter	8 (4.6)	0 (0)	8 (4.6)	.21
Lesser trochanter	2 (1.0)	1 (2.8)	1 (0.6)	.32
Subtrochanteric	6 (2.9)	3 (8.3)	3 (1.7)	.07
ESI Score				.57
2	125 (59.8)	25 (69.4)	100 (57.8)	
3	78 (37.3)	11 (30.6)	67 (38.7)	
4	3 (1.4)	0	3 (1.7)	
5	1 (0.5)	0	1 (0.6)	
NEDOCS score	129 (155)	118 (87.5)	131 (51)	0.19
Mode of arrival				0.02
EMS	185 (88.5)	36 (100)	149 (86.1)	
Walk-in	24 (11.5)	0 (0)	24 (13.9)	
Comorbidities				
Hypertension	91 (43.5)	10 (27.8)	81 (46.8)	.04
Diabetes	76 (36.4)	15 (41.7)	61 (35.3)	.47
Congestive Heart Failure	16 (7.7)	2 (5.6)	14 (8.1)	.60
Chronic Kidney Disease	23 (11.0)	4 (11.1)	19 (11.0)	.98
Cancer	8 (3.8)	1 (2.8)	7 (4.1)	.72

* Chi-square, Fisher exact, and Wilcoxon rank-sum tests were performed.

*ESI*, Emergency Severity Index; *NEDOCS*, National Emergency Department Overcrowding Scale; *EMS*, emergency medical services; *IQR*, interquartile range.

**Table 3:** Description of the type of nerve blocks performed, who performed the nerve block and any associated complications.

Variable	N (%)
Nerve block	
Fascia Iliaca	27 (75)
PENG	7 (19.4)
Femoral	0 (0)
Not specified	2 (5.6)
Who performed the block?	
ED Resident	28 (77.7)
ED Attending	0
ED US Fellow	2 (5.6)
ED US Resident	6 (16.7)
Complications	
Yes	1 (2.8)
No	35 (97.2)

*PENG*, pericapsular nerve group; *ED*, emergency department; *US*, ultrasound.

## Data Availability

Data is available upon request from the corresponding author.
