# Supplementary material for: Nerve Blocks for Hip Fractures in the Emergency Department: An Opportunity for Growth
Source: West J Emerg Med. 2025 Sep 25;26(5):1478–84. doi: 10.5811/westjem.43500 (PMC12591653; doi:10.5811/westjem.43500)
Supplement: Supplementary file 1 [file wjem-26-1478-s001.docx]

1. Click on the appropriate form (Abstractor 1 or 2)
   1. LK is abstractor 1
   2. DB is abstractor 2
2. Automatically assigned, do not change:
   1. Record ID
   2. MRN
   3. FIN
   4. ED visit date, time
3. Exclusion criteria:
   1. Age (automatically calculated from DOB and ED date)
      1. DOB
         1. Pull from chart
      2. ED date (copy from ED visit date)
   2. Pregnant
      1. Take from: ED note (exclude if patient noted to be pregnant) or lab testing (urine or blood)
   3. Prisoner
      1. Exclude (mark yes) if room location listed as Jail or if noted that patient in custody (either nursing/triage or ED note); (hint may be if confidential warning but may be confidential for other reasons)
   4. Transfer from outside hospital
      1. Exclude (mark yes) if ED provider/triage note reports transfer from outside
   5. Contraindication to block
      1. Exclude (mark yes):
         1. if intubated in ED,
         2. Has significant polytrauma: other long bone injury, open fracture, 2+ rib fractures, requires chest tube, other significant distracting injury
         3. AMS or significant intoxication (per ED note)
   6. **If any exclusion criteria (Age<18, no acute hip fx, pregnant, prisoner, transfer, hemodynamic instability, contraindication) do not proceed further**
4. Inclusion criteria:
   1. Hip fracture (acute)
      1. Defined as: needs to be acute, diagnosed in ED
      2. Exclude hip fractures that are non-acute (e.g. sub-acute, chronic, if noted either in radiology study or ED note.) if discrepancy (e.g. xr says indeterminate and CT says acute, go with CT). If discrepancy between ED note and radiology imaging (e.g. radiology note says correlate clinically for acuity, go based off of ED note. If unclear, use best judgement (e.g. if patient presents with fall today, include as acute)
      3. Exclude femoral shaft/diaphysis fractures, acetabular fractures, pathologic fractures.
      4. **If no, stop here**
5. Demographics
   1. Sex
      1. Take from chart “Sex on ID”
   2. Ethnicity
      1. Take from: Demographics>Ethnicity
   3. Race:
      1. Take from Demographics>Race
   4. Language
      1. Take from EHR “Lang/Inter req”
6. ED visit
   1. Mode of arrival:
      1. Taken from ED triage note
         1. EMS
         2. Private vehicle
   2. TTA
      1. Taken from either ED provider or nursing note
   3. ED attending name
      1. Attending who co-signed resident ED note or if was primary
   4. ED resident name
      1. Resident who wrote initial ED note (excluding MSE or signout note)
      2. If patient was pending imaging (hip xr or CT) at time of initial note, can use signout note
   5. Comorbidities
      1. If admitted using admitting H&P or discharge note, if only ED note, use ED provider note
      2. List all conditions
      3. H&P -> ED note→Discharge
   6. Hip fracture type:
      1. Use imaging note, if not available then can use orthopedic consult note
   7. Method of diagnosis:
      1. Select all that apply, all that occurred in the ED
   8. Other traumatic injuries:
      1. Use Admitting and discharge note
         1. If discrepancy (listed on admitting but not discharge, be conservative by listing all)
   9. Non-traumatic diagnoses:
      1. Only if diagnosed in ED (e.g don’t include UTI diagnosed on HD4), use admitting note
   10. Disposition:
       1. Use ED provider or nursing note
   11. Admitting service:
       1. List service who wrote initial admission note
7. Nerve block:
   1. Was a nerve block performed?
      1. Yes if evidence nerve block was done (either by procedure note, ED note, or admitting H&P)
   2. Who performed block?
      1. resident/fellow/attending who wrote procedure note unless otherwise specified (nerve block performed by.) Include resident and cosign attending
   3. Was US fellowship trained person present
      1. Yes if stated it note (procedure, or provider) or if attending co-signer is fellowship trained
      2. Verify with PI if unsure if person is US fellowship trained or not
   4. Any serious complication:
      1. If specifies LAST (local anesthetic systemic toxicity), or if ED or admitting note mentions complication from nerve block
