# Supplementary material for: Nerve Blocks for Hip Fractures in the Emergency Department: An Opportunity for Growth
Source: West J Emerg Med. 2025 Sep 25;26(5):1478–84. doi: 10.5811/westjem.43500 (PMC12591653; doi:10.5811/westjem.43500)
Supplement: Supplementary file 2 [file wjem-26-1478-s001.pdf]

Data Dictionary Codebook

Rate of hip fractures_data collection_Dainis_main (PID: 13296)

28/10/2024 1:30pm

	#	Variable / Field Name	Field Label *Field Note*	Field Attributes (Field Type, Validation, Choices, Calculations, etc.)
**Instrument: Abstractor 1 (abstractor_1)**				
	1	[record_id]	record ID<br>*01, 02, 03, ...*	text
	2	[abstractor]	Abstractor	radio, Required<br>1 Dainis<br>2 Lydia
	3	[mrn]	MRN	text, Required, Identifier
	4	[fin]	FIN	text, Required, Identifier
	5	[ed_visit_date_and_time]	ED visit date and time	text
	6	[date]	ED date (copy from above)	text (date_mdy)
	7	[dob]	DOB	text (date_mdy), Identifier
	8	[age]	Age	calc<br>Calculation: datediff([dob],[date],'y')
	9	[pregnant]	Pregnant<br>*on date of ED visit*	yesno<br>1 Yes<br>0 No
	10	[prisoner]	Prisoner<br>*at time of ED visit*	yesno<br>1 Yes<br>0 No
	11	[transfer]	Received as transfer from outside hospital?	yesno<br>1 Yes<br>0 No
	12	[contraindication]	Contraindication to receiving block (significant polytrauma, intubated, known allergy to local anesthetic)	yesno<br>1 Yes<br>0 No
	13	[contraindication_comment]<br>Show the field ONLY if:<br>[contraindication] = '1'	If contraindication: briefly explain what	text
	14	[exclusion]	Is there an exclusion criteria listed above (age< 18, pregnant, prisoner, transfer, contraindication)? If yes, stop here, do not proceed further	yesno<br>1 Yes<br>0 No
	15	[hip_fx]	Hip fracture (acute, diagnosed in ED). If no, do not proceed further with survey.<br>*need to be acute, diagnosed in ED*	yesno<br>1 Yes<br>0 No

	16	[hip_fx_comment]<br><br>Show the field ONLY if:<br>[hip_fx] = '0'	If no acute hip fx, briefly explain why	text
	17	[hip_fracture_type]<br><br>Show the field ONLY if:<br>[hip_fx] = '1'	Hip fracture type	checkbox<br>1 hip_fracture_type___1 Femoral neck<br>2 hip_fracture_type___2 Intertrochanteric<br>3 hip_fracture_type___3 Femoral head<br>4 hip_fracture_type___4 Greater trochanter<br>5 hip_fracture_type___5 Lesser trochanter<br>6 hip_fracture_type___6 Subtrochanteric
	18	[imaging]<br><br>Show the field ONLY if:<br>[hip_fx] = '1'	Method of diagnosis (select all that were done in ED)<br>*at time of ED visit*	checkbox<br>1 imaging___1 X-ray<br>2 imaging___2 CT<br>3 imaging___3 MRI<br>4 imaging___4 Other
	19	[other_hip_fx_dx]<br><br>Show the field ONLY if:<br>[imaging(4)] = '1'	Unknown method of diagnosis	text
	20	[sex]	Sex	checkbox<br>1 sex___1 Male<br>2 sex___2 Female
	21	[ethnicity]	Ethnicity	radio<br>1 Hispanic or latino<br>2 Non-latino<br>3 Unknown
	22	[race]	Race	checkbox<br>1 race___1 White<br>2 race___2 Black or African American<br>3 race___3 Asian<br>4 race___4 Native Hawaiian or Other Pacific Islander<br>5 race___5 American Indian or Alaska Native<br>6 race___6 Other<br>7 race___7 Unknown
	23	[race_other]<br><br>Show the field ONLY if:<br>[race(6)] = '1'	Race other	text
	24	[language]	Language	checkbox

				1 language___1 English<br>2 language___2 Spanish<br>3 language___3 Mandarin<br>4 language___4 Korean<br>5 language___5 Other
	25	[language_other]<br>Show the field ONLY if:<br>[language(5)] = '1'	Language other	text
	26	[arrival_method]	Mode of arrival	dropdown<br>1 EMS<br>2 Walk-in/private vehicle<br>3 Other
	27	[mode_of_arrival_other]	Mode of arrival other	text
	28	[tta]	TTA<br>*trauma team activation*	yesno<br>1 Yes<br>0 No
	29	[ed_attending]	ED attending name (co-signer of ED note)	text
	30	[ed_resident]	ED Resident name (author of ED note)	text
	31	[comorbidities]	Comorbidities<br>*Check all*	checkbox<br>1 comorbidities___1 Hypertension<br>2 comorbidities___2 Diabetes<br>3 comorbidities___3 Heart Failure<br>4 comorbidities___4 Chronic kidney disease<br>5 comorbidities___5 Cancer<br>6 comorbidities___6 Other
	32	[other_comorbid]<br>Show the field ONLY if:<br>[comorbidities(6)] = '1'	Comorbidities (other)	text
	33	[other_trauma]	Other traumatic injuries<br>*list all acute traumatic injuries diagnosed in ED*	text
	34	[non_trauma_ed_diagnoses]	Non-traumatic ED diagnoses<br>*only if diagnosed in ED, list all*	text
	35	[dispo]	Disposition	checkbox<br>1 dispo___1 Admitted<br>2 dispo___2 Observation<br>3 dispo___3 Transferred<br>4 dispo___4 Died in ED<br>5 dispo___5 Discharged

				6 | dispo___6 | AMA<br>7 | dispo___7 | Unknown<br>8 | dispo___8 | Other
	36	[transfer_location]<br><br>Show the field ONLY if:<br>[dispo(3)] = '1'	Transferred location<br>*Name of accepting hospital*	text
	37	[admitting_service]<br><br>Show the field ONLY if:<br>[dispo(1)] = '1' and [disp o(2)] = '1'	Admitted service	radio<br>1 | Orthopedics<br>2 | Trauma<br>3 | Medicine<br>4 | Other
	38	[nerve_block]	Nerve block?<br>*for hip fx*	yesno<br>1 | Yes<br>0 | No
	39	[nerve_block_proof]<br><br>Show the field ONLY if:<br>[nerve_block] = '1'	How was block identified?	checkbox<br>1 | nerve_block_proof___1 | Procedure note<br>2 | nerve_block_proof___2 | ED note<br>3 | nerve_block_proof___3 | Nursing note<br>4 | nerve_block_proof___4 | Other
	40	[nb_other]<br><br>Show the field ONLY if:<br>[nerve_block_proof(4)] = '1'	Nerve block other	text
	41	[block]<br><br>Show the field ONLY if:<br>[nerve_block] = '1'	Who performed block? (include attending in addition to resident who wrote note)	checkbox<br>1 | block___1 | ED attending<br>2 | block___2 | ED resident<br>3 | block___3 | ED US fellow<br>4 | block___4 | ED US team<br>5 | block___5 | non-ED (anesthesia, trauma)<br>6 | block___6 | other<br>7 | block___7 | unknown
	42	[us_fellow]	Was US fellowship trained person present?<br>*US fellows included, must be mentioned somewhere in note*	yesno<br>1 | Yes<br>0 | No
	43	[block_name]<br><br>Show the field ONLY if:<br>[nerve_block] = '1'	Name(s) of who performed block<br>*List all*	text
	44	[nerve_block_type]	Nerve block type	checkbox

		Show the field ONLY if: [nerve_block] = '1'		1 nerve_block_type___1 Femoral<br>2 nerve_block_type___2 Fascia iliaca<br>3 nerve_block_type___3 PENG<br>4 nerve_block_type___4 Not specified
	45	[serious_complication]	Any serious complications (e.g. LAST) of nerve block?	yesno<br>1 Yes<br>0 No
	46	[serious_complication_desc]	serious complication description	text
	47	[comments]	comments<br>*list any additional comments*	text
	48	[abstractor_1_complete]	Section Header: *Form Status*<br>Complete?	dropdown<br>0 Incomplete<br>1 Unverified<br>2 Complete
